# Supplementary material for: MBP-11901 Inhibits Tumor Growth of Hepatocellular Carcinoma through Multitargeted Inhibition of Receptor Tyrosine Kinases
Source: Cancers (Basel). 2022 Apr 14;14(8):1994. doi: 10.3390/cancers14081994 (PMC9030223; doi:10.3390/cancers14081994)
Supplement: Supplementary file 1 [file cancers-14-01994-s001.zip › cancers-1659720-supplementary.pdf]

cancers

MDPI

Article

MBP-11901 Inhibits Tumor Growth of Hepatocellular Carcinoma through Multitargeted Inhibition of Receptor Tyrosine Kinases

Hyun Jin Park [1,†], Garam Choi [1,†], Seongmin Ha [2], Yesl Kim [1], Min-Jin Choi [1], Minsup Kim [3], Md. Kamrul Islam [2], Yongmin Chang [2], Tae-Jun Kwon [4], Dongkyu Kim [4], Eunbee Jang [5], Tae Hwan Kim [5], Sha Joung Chang [1] and Yeoun-Hee Kim [1,*]

[1] R&D Center, Etnova Therapeutics Corp., 124, Sagimakgol-ro, Jungwon-gu, Seongnam-si 13207, Korea; phj0808@etnova.co.kr (H.J.P.); garam1458@etnova.co.kr (G.C.); yesl_kim@etnova.co.kr (Y.K.); zzz9924@etnova.co.kr (M.-J.C.); sjchang3136@etnova.co.kr (S.J.C.)
[2] Institute of Biomedical Engineering Research, Kyungpook National University, 680, Gukchaebosang-ro, Jung-gu, Daegu 41944, Korea; 996574@naver.com (S.H.); mkislam2008@yahoo.com (M.K.I.); ychang@knu.ac.kr (Y.C.)
[3] InCerebro Drug Discovery Institute, Seoul 01811, Korea; minsupkim.bio@gmail.com
[4] Preclinical Research Center, Daegu-Gyeongbuk Medical Innovation Foundation, 80, Cheombok-ro, Dong-gu, Daegu 41061, Korea; tjkwon@kmedihub.re.kr (T.-J.K.); dgkim728@kmedihub.re.kr (D.K.)
[5] College of Pharmacy, Daegu Catholic University, 13-13, Hayang-ro, Hayang-eup, Gyeongsan-si 38430, Korea; wkddmsql96@naver.com (E.J.); thkim@cu.ac.kr (T.H.K.)
* Correspondence: yhkim@etnova.co.kr; Tel.: +82-31-776-3403
† These authors contributed equally to this work.

A

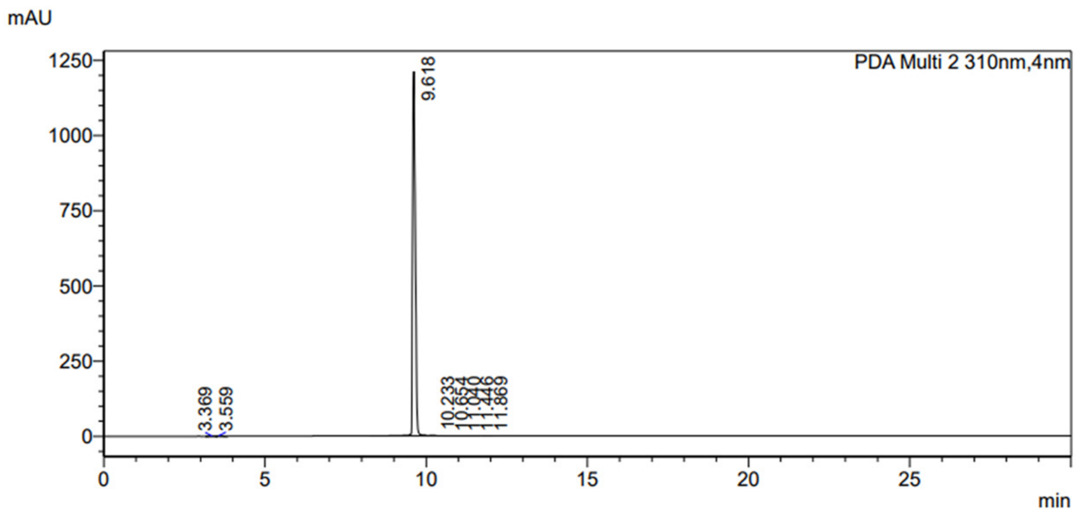

B

Peak#	Ret. Time	Area	Height	%Area
1	3.369	2468	209	0.03
2	3.559	8813	1264	0.12
3	9.618	7254657	1211858	99.64
4	10.233	6443	976	0.09
5	10.654	3464	380	0.05
6	11.040	1735	232	0.03
7	11.446	1666	201	0.02
8	11.869	1716	242	0.02
Total		7280962	1215360	100%

Figure S1. HPLC analysis data of MBP-11901.

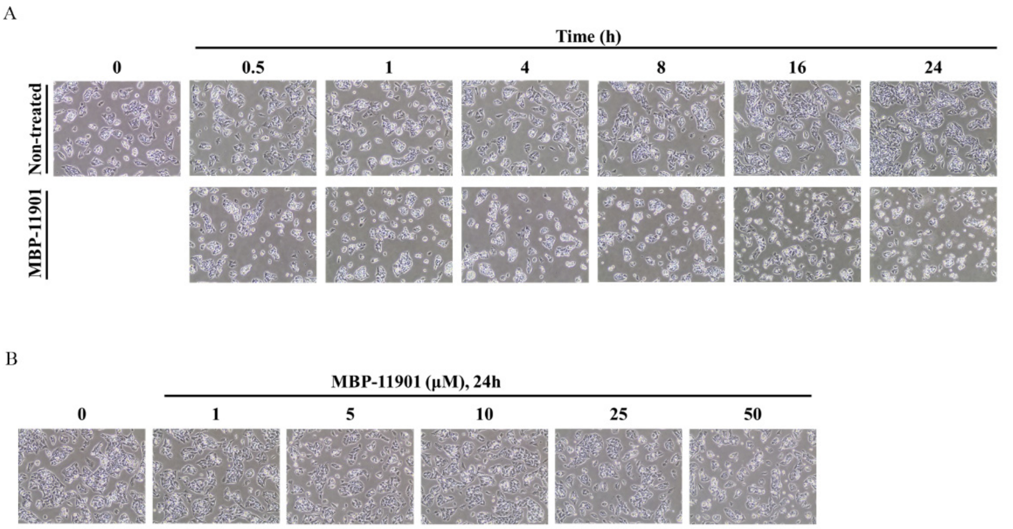

Figure S2. Morphological changes of HepG2 cells after exposure to MBP-11901. HepG2 cells were exposed to 50 μM MBP-11901 for various times (0.5, 1, 4, 8, 16, and 24 h) (A) and different concentrations of MBP-11901 for 24 h (B).

.

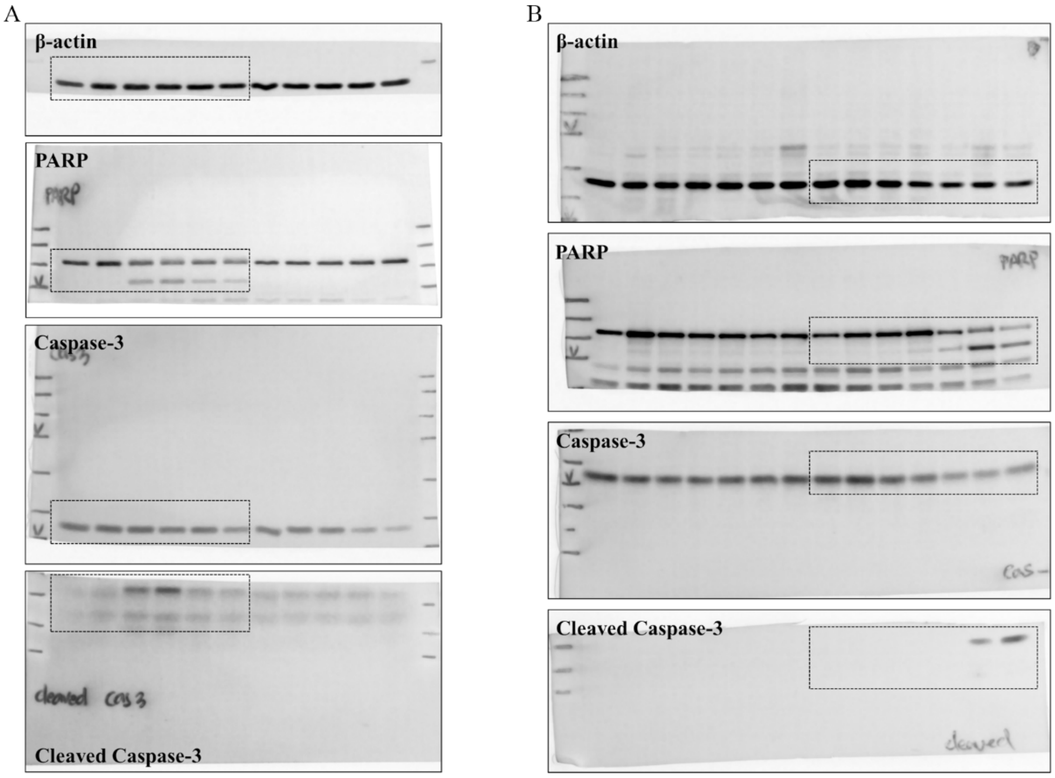

Figure S3. Source data of western blot analysis shown in Figure 2A and 2B.

Table S1. IC_{50} values of MBP-11901 after treatment of various normal and cancer cell lines for 24 h.

Tissue sources	Cell lines	IC_{50} (μM) for MBP-11901
Normal Cell Lines	FHC	72.74±1.99
	MCF10a	65.13±1.88
	HEK-293	68.15±2.79
	NE-4C	36.82±3.66
	AML12	137.65±24.15
Cancer Cell Lines	Caki-2	75.65±3.44
	PC3	95.00±0.82
	A549	100.16±8.44
	MDA-MB-231	62.02±6.5

	HepG2	5.16±1.37
	HeLa	41.91±3.38
	C6	77.84±9.73
	MCF-7	65.94±12.34
	HT-29	29.86±0.27

Table S2. IC_{50} values of MBP-11901 or sorafenib after treatment of normal and various hepatic carcinoma cell lines for 24 h.

Tissue sources	Cell lines	MBP-11901		Sorafenib	
		IC_{50} (μM)	R^2	IC_{50} (μM)	R^2
Normal Cell Lines	AML12	65.70 ± 1.32	0.96	13.44 ± 2.26	0.91
Hepatocarcinoma Cell Lines	HepG2	5.16 ± 1.12	0.85	3.85 ± 0.14	0.97
	Hep3B	16.82 ± 0.08	0.98	3.64 ± 0.99	0.97
	Huh-7	29.41 ± 0.92	0.98	6.47 ± 0.88	0.97
	PLC/PRF.5	18.55 ± 0.66	0.91	6.81 ± 0.76	0.98

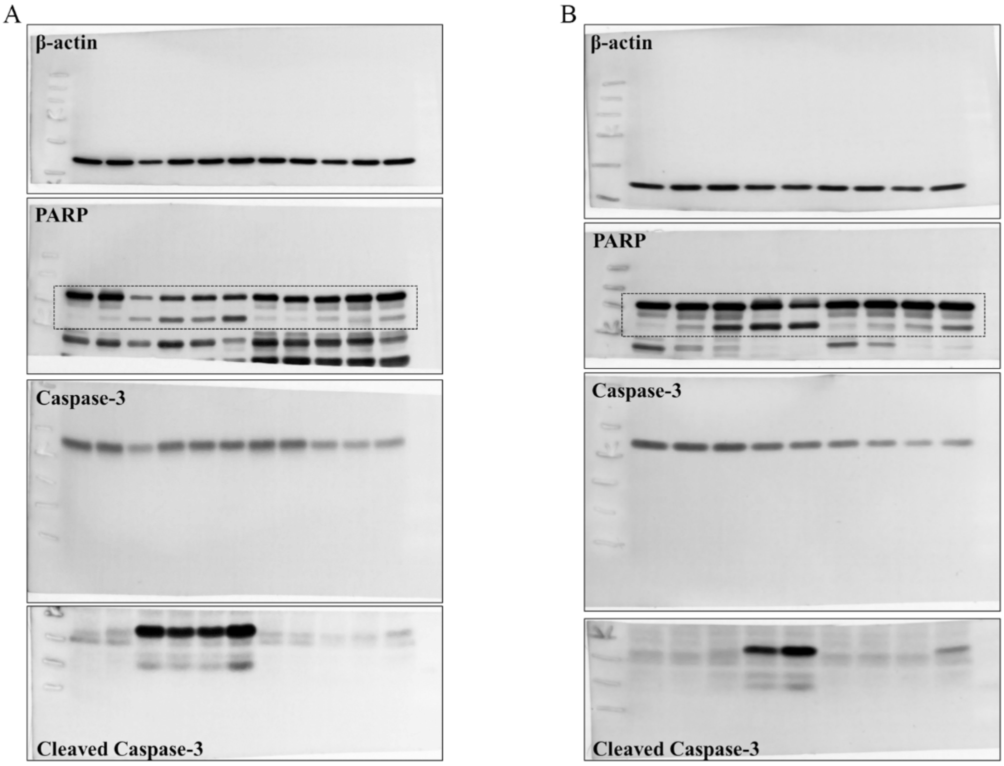

Figure S4. Source data of western blot analysis shown in Figure 3D and 3E.

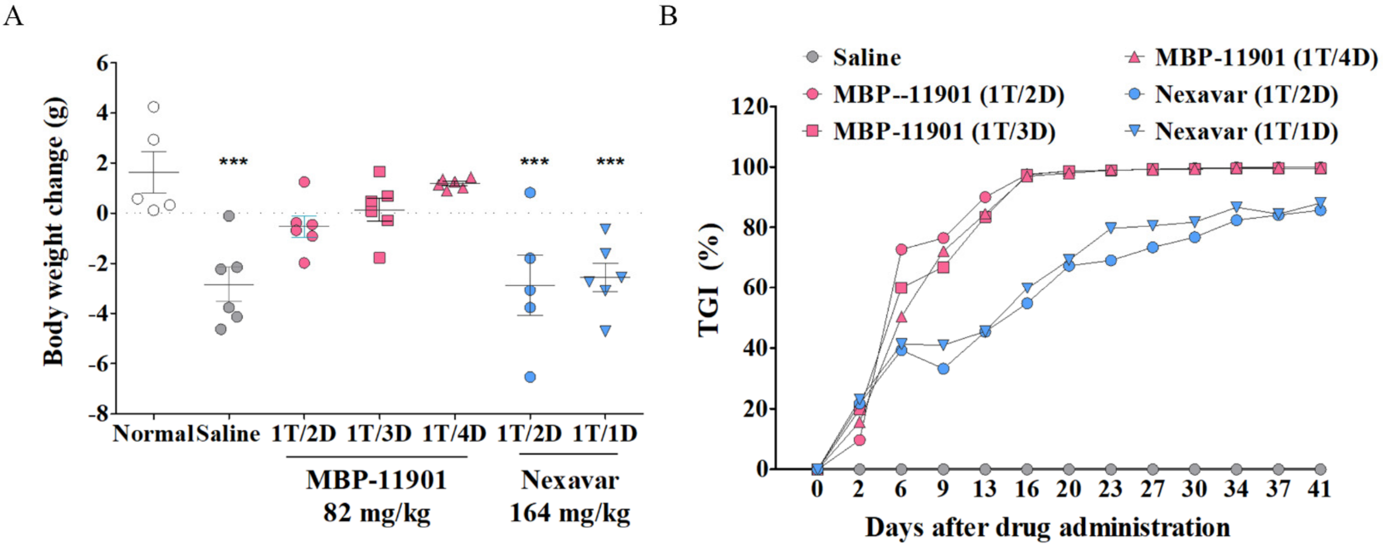

Figure S5. Final body weight of animals excluding increased tumor weight and TGI (%). The actual body weight except for the increased tumor weight at the end of the administration according to the intervals of oral administration of MBP-11901 (A). Graph of TGI (%) analysis shown in Table 4 (B).

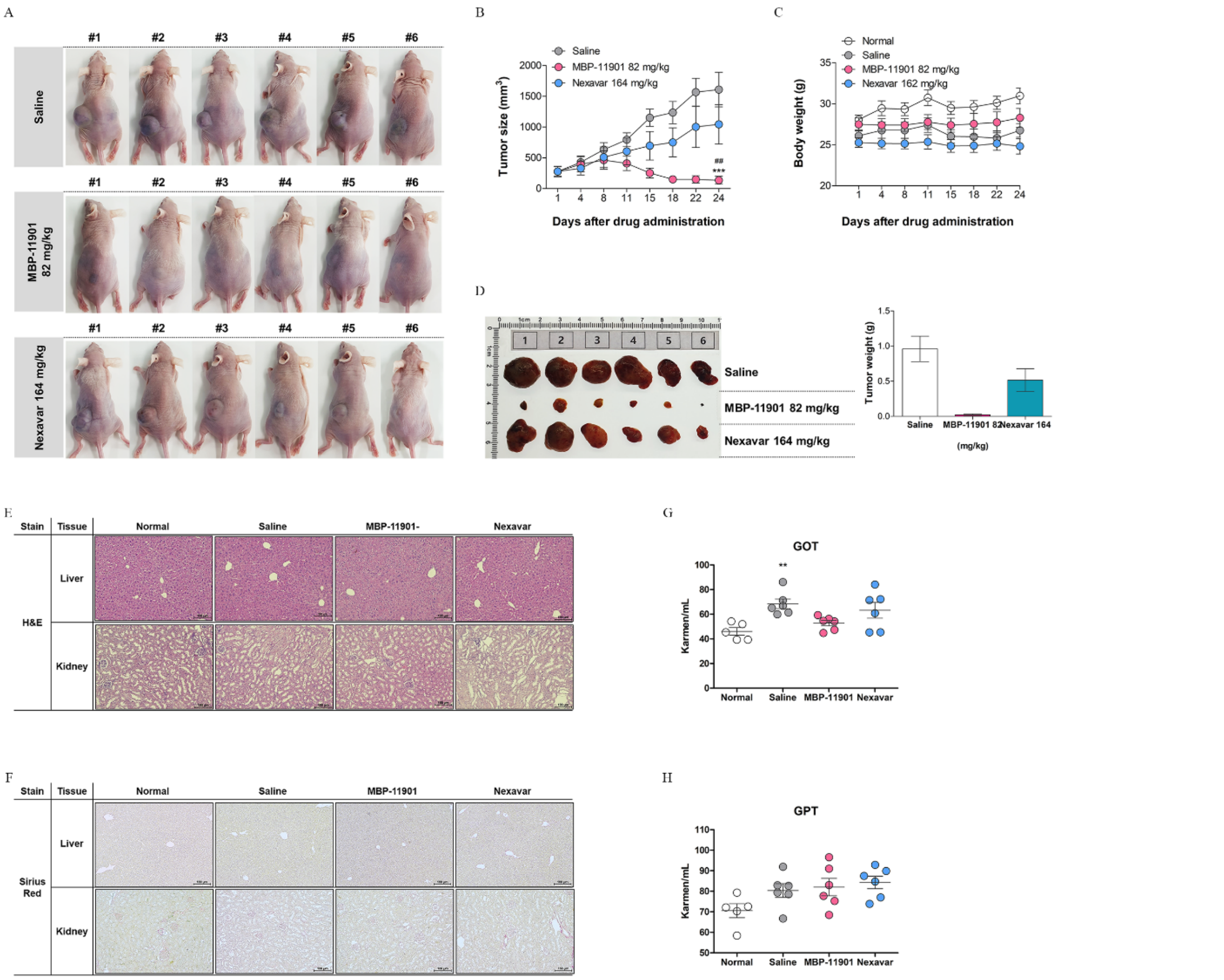

Figure S6. Anticancer effect of MBP-11901 in the Hep3B HCC cell line. (A) Images of mice in each group at the end of the experiment. Tumor volume (B) and body weight (C) of mice in each group. (D) Tumor weight, (E, F) H&E and Sirius red staining of liver and kidney tissues, as well as levels of GOT (G) and GPT (H).

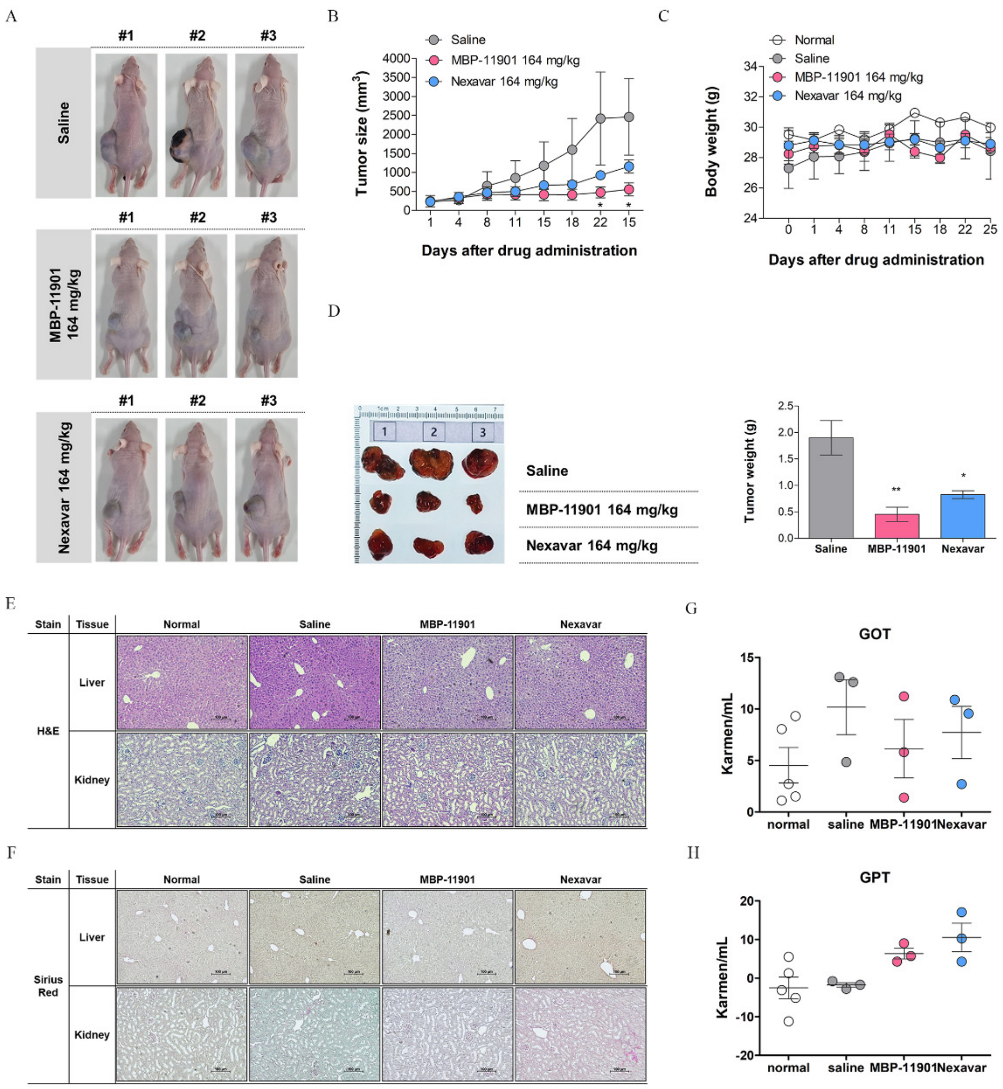

Figure S7. Anticancer effect of MBP-11901 in the Huh-7 HCC cell line. (A) Images of mice in each group at the end of the experiment. Tumor volume (B) and body weight (C) of mice in each group. (D) Tumor weight, (E, F) H&E and Sirius red staining of liver and kidney tissues, as well as levels of GOT (G) and GPT (H).

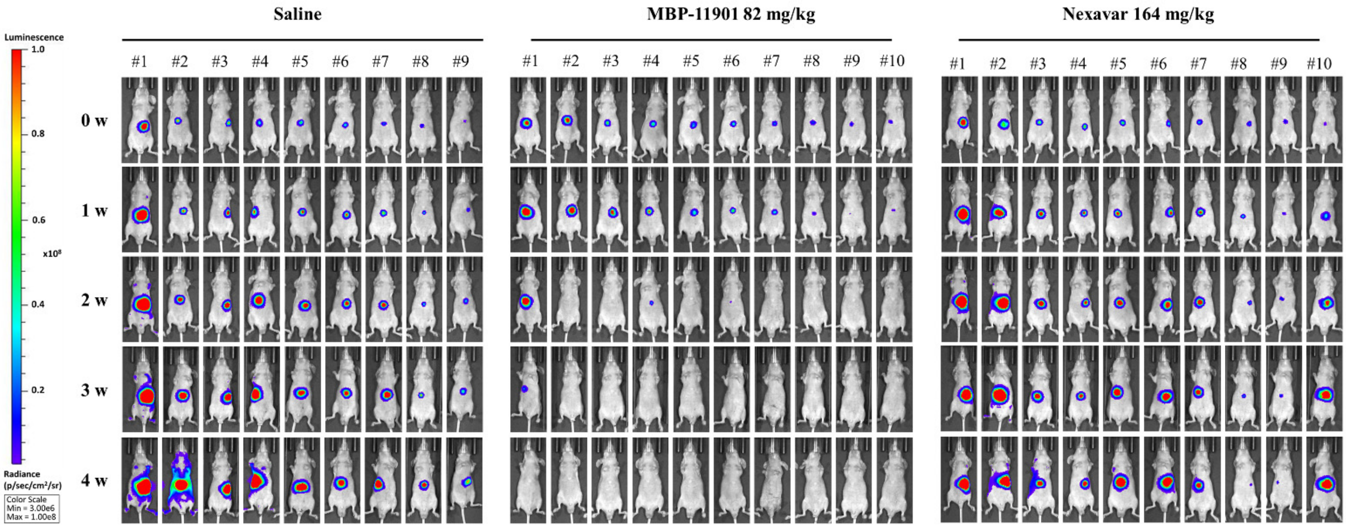

Figure S8. Imaging data of efficacy evaluation of MBP-11901 performed using an orthotopic animal model.

Table S3. Ratio of the average luminescence intensity of mice in each group to the average luminescence intensity of those in the saline group, immediately before oral administration of drugs.

Group	Ratio				
	0 w	1 w	2 w	3 w	4 w
Saline	1.00 ± 1.8	4.55 ± 10.1	10.80 ± 20.7	13.77 ± 23.3	26.92 ± 30.3
MBP-11901	0.77 ± 0.8	2.24 ± 2.9	0.83 ± 1.8	0.03 ± 0.1	0.02 ± 0.0 *
Nexavar®	0.82 ± 0.9	4.73 ± 6.9	9.05 ± 11.2	9.49 ± 10.9	11.69 ± 10.0

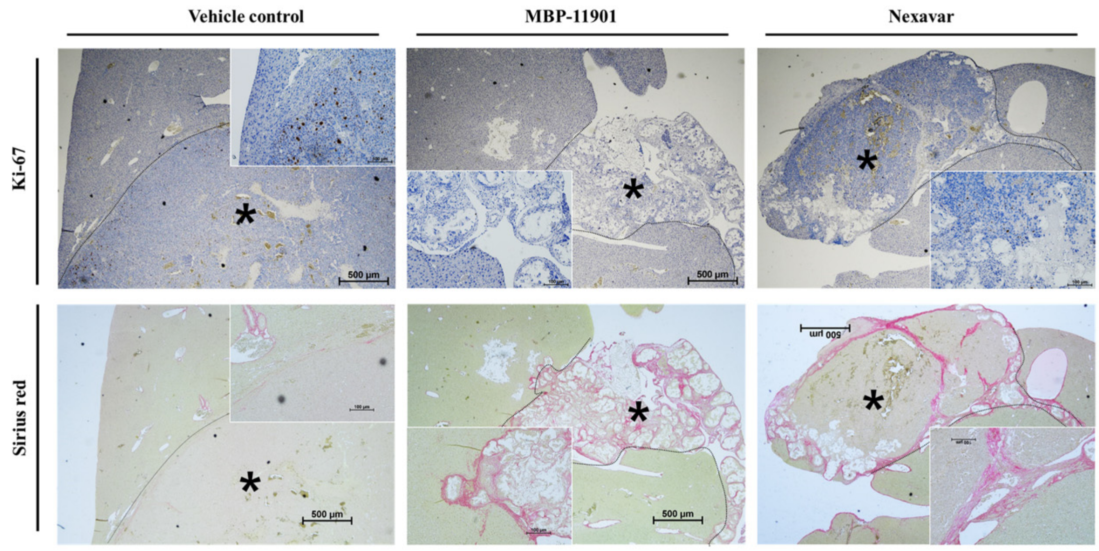

Figure S9. Proliferation marker (antibody Ki-67) and Sirius red staining in resected transplanted tumors from the HepG2 orthotopic transplantation model.

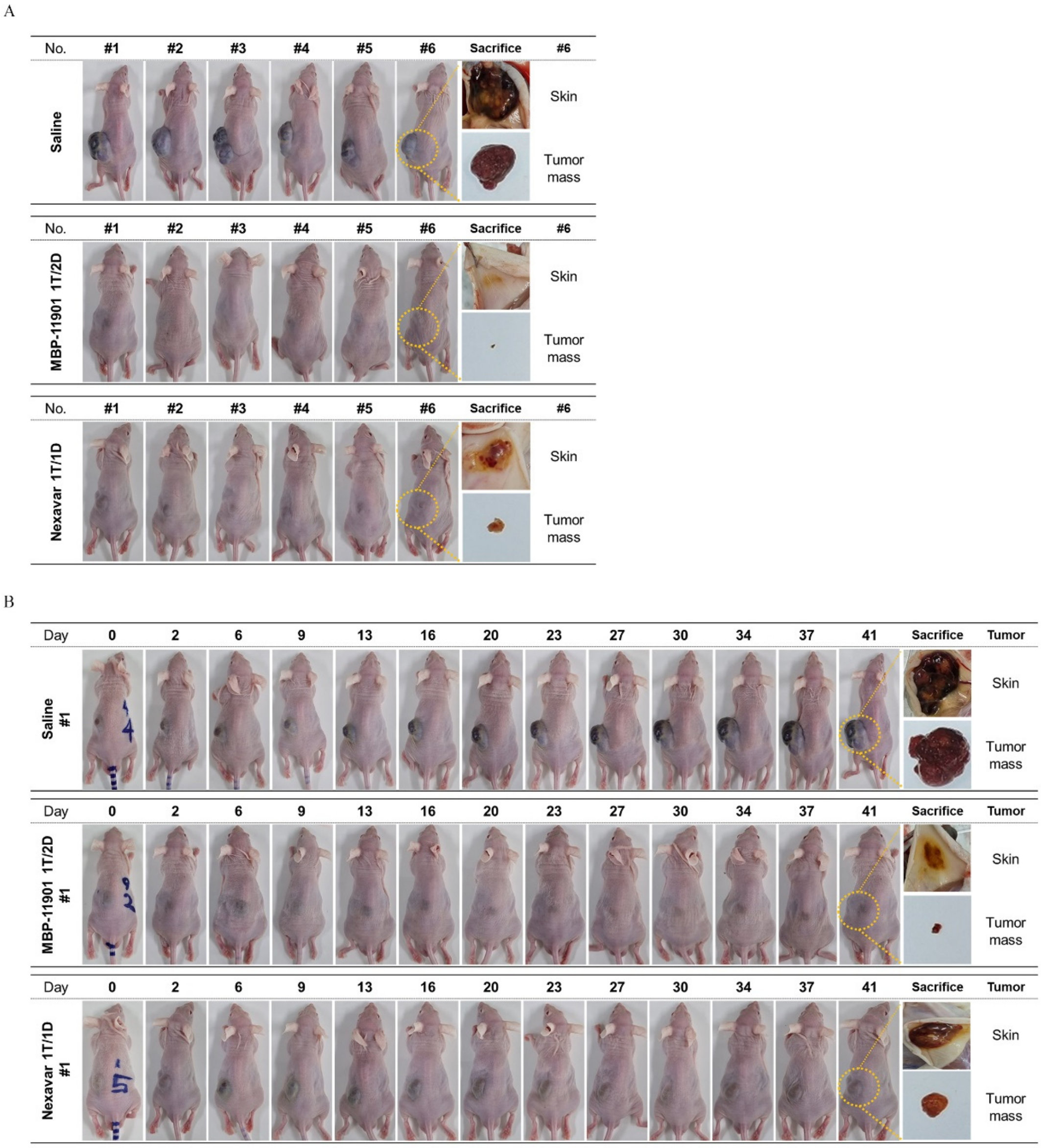

Figure S10. Images of subcutaneously implanted tumor sites according to oral administration interval. (A) Images of tumors inside the skin of mice in each group; and resected tumor from the last animal in each group. (B) Images of the first animal in each group according to date; images of tumors inside the skin at the end of administration; and images of resected tumors.

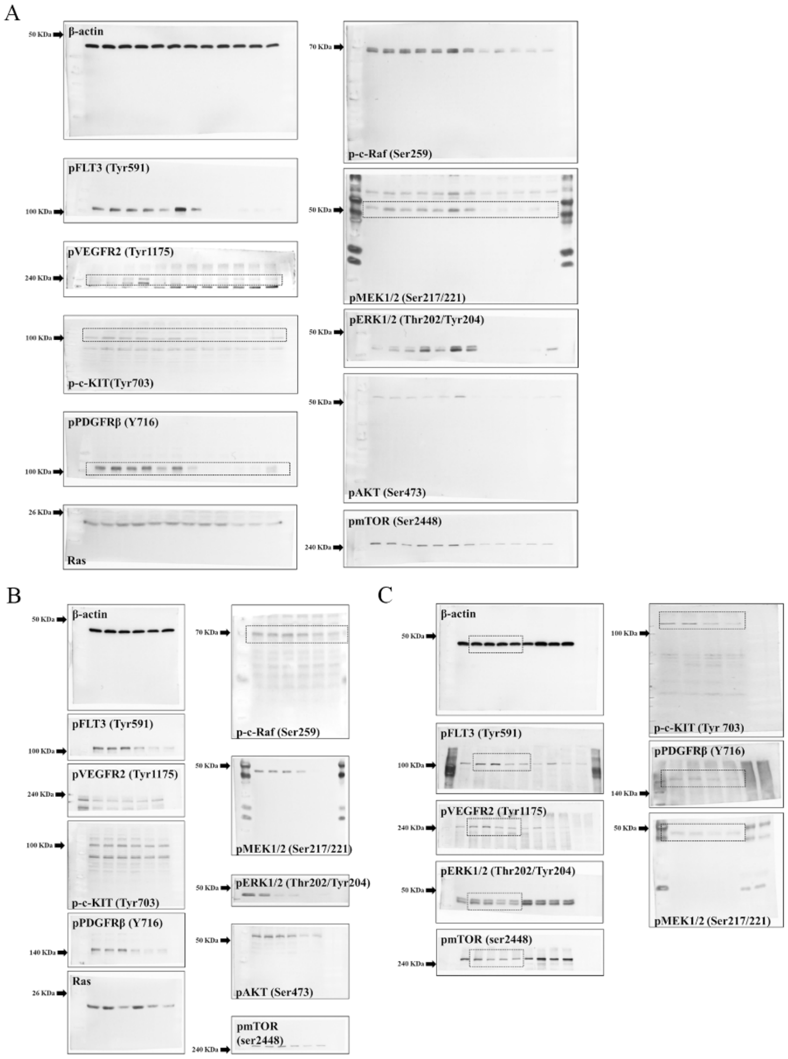

Figure S11. Source data of western blot analysis shown in Figure 7C, D and E.

Target	Raf	MEK1	MEK2	ERK1	ERK2
**2D**	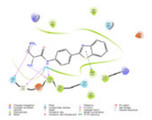	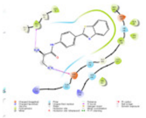	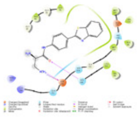	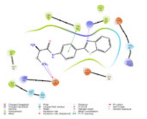	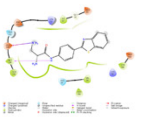
**3D**	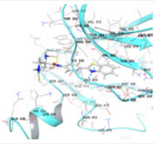	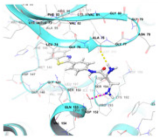	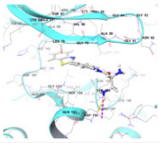	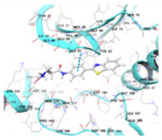	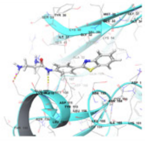
**Score (Kcal/mol)**	**-6.963**	**-7.331**	**-6.142**	**-5.983**	**-6.889**

Figure S12. 2D and 3D ligand interaction of MBP-11901 and Raf/MEK/ERK signal-related target proteins.
